# MsDAD1 acts as a heat-induced “senescence brake” in alfalfa

**DOI:** 10.3389/fpls.2025.1664465

**Published:** 2025-09-05

**Authors:** Yuguang Song, Xinying Guo, Linyao Wang, Yingying Zheng, Ting Li, Wei Dong

**Affiliations:** School of Life Sciences, Qufu Normal University, Qufu, Shandong, China

**Keywords:** MsDAD1, heat stress, senescence, flowering, *Medicago sativa*

## Abstract

Heat stress severely limits the productivity of alfalfa (*Medicago sativa* L.). In this study, the defender against apoptotic death 1 (*DAD1*) gene, *MsDAD1*, was identified and functionally characterized as a key positive regulator of heat tolerance. The expression of *MsDAD1* was specifically and strongly induced by heat stress, and phylogenetic analysis confirmed its high conservation across plant species. Ectopic overexpression of *MsDAD1* in transgenic alfalfa significantly enhanced tolerance to heat stress. Compared to wild-type plants, *MsDAD1*-overexpressing lines (*MsDAD1*-OE) exhibited reduced leaf chlorosis and abscission, higher relative water content, lower electrolyte leakage, greater chlorophyll retention, and diminished accumulation of reactive oxygen species (H_2_O_2_, O_2_^-^) and malondialdehyde (MDA), suggesting improved membrane integrity and reduced oxidative damage. Transcriptome (RNA-seq) analysis and subsequent physiological validation indicated that *MsDAD1* suppresses heat-induced accumulation of jasmonic acid (JA) and abscisic acid (ABA) by down-regulating key biosynthetic genes, *LOX1* and *NCED1*. As a result, *MsDAD1*-OE plants displayed attenuated JA- and ABA-mediated leaf senescence under heat stress. Furthermore, *MsDAD1* overexpression delayed heat-induced flowering, correlating with the repression of flowering-promoting genes such as *FT* and *ELF4*. Collectively, these findings demonstrate that *MsDAD1* enhances alfalfa heat tolerance by mitigating oxidative stress, modulating JA and ABA biosynthesis to delay senescence, and altering flowering time under high-temperature conditions. *MsDAD1* represents a promising genetic target for improving heat resilience in alfalfa.

## 1 Introduction

Temperature is a critical environmental factor influencing plant growth, development, geographical distribution, quality, and productivity (Zhou et al., 2024). Global temperatures are projected to rise by approximately 0.2°C per decade, potentially reaching 1.8–4.0°C above current levels by the year 2100. Recent studies have shown that for each 1°C increase in average temperature, yields of major crops such as wheat, rice, maize, and soybeans decline by 6.0%, 3.2%, 7.4%, and 3.1%, respectively (Arshad and Hannoufa, 2022; Battisti and Naylor, 2009; Zhao et al., 2017). Heat stress induces a wide range of often detrimental changes in plant growth, morphology, physiological processes, and ultimately yield (Arshad and Hannoufa, 2022). At the cellular and biochemical levels, heat stress disrupts metabolite homeostasis and inhibits numerous physiological and biochemical processes. These include alterations in water status, membrane stability, photosynthesis, secondary metabolite production, and hormone balance (Guo et al., 2023). Heat stress rapidly impairs photosynthesis by damaging chloroplast ultrastructure, reducing photosynthetic pigment content, and impairing photosystem II function (Li et al., 2018). Additionally, it affects protein synthesis and stability, compromises membrane integrity, and induces the accumulation of reactive oxygen species (ROS) (Correia et al., 2022). Heat stress also disrupts organelle function, alters hormone signaling pathways (Ding and Yang, 2022), and perturbs calcium and lipid signaling as well as kinase activity (e.g., MAPKs, CBKs, CDPKs). These responses are accompanied by transcriptomic reprogramming and widespread metabolomic shifts (Haider et al., 2021).

The plant hormone jasmonic acid (JA) plays a critical role in the response to heat stress. Studies have shown that heat stress induces the accumulation of JA and its derivatives, including jasmonoyl-isoleucine (JA-Ile) and 12-oxo-phytodienoic acid (OPDA), thereby enhancing cell viability and heat tolerance in *Arabidopsis* (Wang et al., 2023). Exogenous application of JA to wild-type plants prior to heat exposure alleviates heat-induced damage, indicating that JA directly contributes to heat stress protection (Hu et al., 2017). However, excessive JA accumulation can also promote premature senescence in plants. For instance, the expression levels of *LOX1* (*LIPOXYGENASE 1*), *LOX3*, and *LOX4* increase markedly during leaf senescence, resulting in significantly higher JA concentrations in senescent leaves compared to non-senescent ones (Hu et al., 2017). Furthermore, treatment with exogenous JA accelerates leaf senescence and induces the expression of senescence-associated genes (Lim et al., 2007).

In addition to its role in stress responses, jasmonic acid (JA) also plays a critical role in inflorescence and flower development (Yuan and Zhang, 2015). JA has been identified as a key phytohormone regulating diurnal flower-opening time (DFOT) in rice (Zhu et al., 2024). In *Arabidopsis*, peroxisomal β-oxidation enzymes—including ACYL-COA OXIDASE (ACX), MULTIFUNCTIONAL PROTEIN (MFP; possessing 2-trans-enoyl-CoA hydratase and L-3-hydroxyacyl-CoA dehydrogenase activities), and 3-KETOACYL-COA THIOLASE (KAT)—are essential for proper inflorescence patterning (Yuan and Zhang, 2015; Ghasemi Pirbalouti et al., 2014; Schaller and Stintzi, 2009; Wiszniewski et al., 2014). Exogenous application of methyl jasmonate (MeJA) has been shown to promote flowering time and influence floral organ development in oilseed rape (*Brassica napus* L.) (Pak et al., 2009). More recently, MeJA treatment was found to accelerate DFOT in rice, with the proportion of opened florets increasing in a concentration-dependent manner (Wang M et al., 2024). While the roles of JA in plant development and stress adaptation are well established, the molecular mechanisms underlying its regulation of temperature-dependent flowering time and heat-induced leaf senescence remain largely unknown.

The *DAD1* protein was initially identified in the temperature-sensitive tsBN7 mutant cell line (Zhou et al., 1997). DAD1 functions as a subunit of the oligosaccharyltransferase (OST) complex, a key catalytic component of the endoplasmic reticulum (ER) (Wang et al., 2024). The OST complex catalyzes N-glycosylation in the ER, facilitating the attachment of oligosaccharides to specific asparagine residues on nascent polypeptides. This modification is essential for proper protein folding and subsequent export from the ER (Yan et al., 2005; Roboti and High, 2012; Zhang et al., 2016). Although DAD1 family proteins have been implicated in salinity tolerance, high-light responses, and disease resistance in plants, their roles in heat stress regulation remain poorly understood (Wang et al., 2022; Yan et al., 2019; Wang X et al., 2024; Beaugelin et al., 2019).

In this study, we identified *MsDAD1* as a heat-inducible gene in alfalfa. Overexpression of *MsDAD1* suppressed the heat-induced hyperaccumulation of jasmonic acid (JA) and abscisic acid (ABA), enhanced reactive oxygen species (ROS) scavenging, and delayed both leaf senescence and flowering under heat stress. We propose that *MsDAD1* functions as a heat-responsive “senescence brake” in alfalfa, offering a promising genetic target for future breeding efforts aimed at improving thermotolerance.

## 2 Materials and methods

### 2.1 Plant material, growth conditions and stress treatment

The alfalfa (*Medicago sativa* L.) genotypes used in this study included the wild-type cultivar SY4D and *MsDAD1* transgenic lines (OE#1 and OE#3), which were generated in the SY4D background. Rooted stem cuttings of both wild-type and transgenic plants were prepared and transplanted into 10×10 cm pots. Plants were grown under controlled environmental conditions: a 16/8 h light/dark photoperiod, a temperature of 23°C, relative humidity of 50–70%, and a light intensity of 300 μmol m^-^² s^-^¹.

To analyze the expression pattern of *MsDAD1* under heat stress, four-week-old alfalfa seedlings were transferred to a climate chamber and exposed to high-temperature treatment (40°C) for 0, 1, 3, 6, 12, 24, and 48 hours. To evaluate the heat tolerance function of *MsDAD1*, transgenic and wild-type plants were subjected to a controlled heat stress regime in a growth chamber set at 32°C (night)/40°C (day), allowing for plant survival and the assessment of physiological responses. Phenotypic evaluations were conducted after six days of treatment.

For flowering time analysis under both normal and heat stress conditions, above ground part of wild-type and *MsDAD1*-overexpressing (OE) plants were cut off at the same time. Plants were divided into two groups: one maintained under normal conditions (20°C night/23°C day), and the other exposed to a heat stress regime of 30°C (night)/35°C (day), with identical photoperiod and light intensity settings as the control group.

For hormone treatment assays, healthy mature leaves were divided into two groups. One group was placed on half-strength Murashige and Skoog (½ MS) medium (control), and the other was treated with 50 μM jasmonic acid (JA) or abscisic acid (ABA), respectively. Unless otherwise stated, all experiments were performed with at least three biological replicates.

### 2.2 Gene isolation and sequence analysis

The full-length coding sequence (CDS) of *MsDAD1* was amplified using gene-specific primers listed in **Supplementary Table S1**. PCR amplification was performed with an initial denaturation at 98°C for 3 minutes, followed by 35 cycles of 98°C for 10 seconds, 55°C for 30seconds, and 72°C for 1 minute, with a final extension at 72°C for 10 minutes. The amplified product was ligated into the pMD19-T vector (Takara) and verified by Sanger sequencing. Homologous polypeptide sequences of DAD1 were retrieved from the GenBank database. Phylogenetic analysis was conducted using the neighbor-joining method. Sequence alignment was performed with ClustalX (www.clustal.org), and the phylogenetic tree was constructed using MEGA version 6.0 (www.megasoftware.net) with 1000 bootstrap replicates to assess branch support and reliability.

### 2.3 RNA extraction and qRT‐PCR

Total RNA was extracted using the MiniBEST Plant RNA Extraction Kit (TaKaRa, Dalian, China), following the manufacturer’s protocol. First-strand cDNA synthesis was performed using the TransScript II One-Step gDNA Removal and cDNA Synthesis SuperMix Kit (TransGen, Beijing, China). Quantitative real-time PCR (qRT-PCR) was conducted using 2× SYBR Green Mix (Vazyme, Cat. No. Q711-03) according to the manufacturer’s instructions. The *ACT2* gene was used as an internal reference for normalization. Relative transcript levels were calculated using the 2^^–ΔΔCt^ method (Livak and Schmittgen, 2001). Primer sequences are listed in **Supplementary Table S1**. Each biological sample was analyzed in triplicate.

### 2.4 Plasmid construction and genetic transformation

To generate transgenic alfalfa seedlings, the coding sequence of *MsDAD1* was cloned and inserted into the binary vector 35S::NOS::1300, under the control of the CaMV:: 35S promoter. The resulting construct was introduced into alfalfa via *Agrobacterium tumefaciens*-mediated transformation, following the protocol described in *Transgenic Plants: Methods and Protocols* (Jiang et al., 2019). Transgenic lines were selected on hygromycin-containing medium, and successful integration of *MsDAD1* was confirmed by PCR analysis.

### 2.5 Physiological measurement

Relative water content (RWC) was determined using the leaf saturation method. Electrolyte leakage (EL) was measured with a conductivity meter (BELL, BEC-6600, Dalian, China). Briefly, six fully expanded, healthy leaves were collected from the middle canopy of each plant at the same developmental stage. The fresh weight of each sample was recorded prior to analysis and then incubated in 25 mL of double-distilled water. After 2 hours of gentle shaking, the initial conductivity of the solution was measured using a DIST-5 conductometer (Hanna Instruments). Samples were then boiled to release all electrolytes, and the final conductivity was recorded. EL was expressed as a percentage of the total conductivity and normalized to fresh weight. Chlorophyll content was measured using a SPAD chlorophyll meter (SPAD-502; Konica Minolta Sensing, Japan). Hydrogen peroxide (H_2_O_2_), malondialdehyde (MDA), and superoxide anion (O_2_^-^) levels were quantified using commercial assay kits (Jiancheng Bioengineering Institute, Nanjing, China). Endogenous levels of jasmonic acid (JA) and abscisic acid (ABA) were quantified using 20 mg of fresh plant tissue. Phytohormones were extracted with 10% (v/v) methanol in water (MeOH/H_2_O). A cocktail of stable isotope-labeled internal standards was added to validate the liquid chromatography–mass spectrometry (LC-MS) quantification. The extracts were purified using Oasis hydrophilic-lipophilic balanced (HLB) columns (30 mg/1 mL; Waters), and targeted analytes were eluted with 80% (v/v) methanol. The eluent, containing both neutral and acidic compounds, was gently evaporated to dryness under a stream of nitrogen. Chromatographic separation was carried out using an Acquity Ultra Performance Liquid Chromatography (UPLC) system (Waters) equipped with an Acquity UPLC BEH C18 column (100 × 2.1 mm, 1.7 µm; Waters). The effluent was introduced into the electrospray ionization (ESI) source of a Xevo TQ-S triple quadrupole mass spectrometer (Waters) for targeted quantification of JA and ABA.

### 2.6 Transcriptomic analysis

Two-week-old wild-type and *MsDAD1*-overexpressing (OE) seedlings were cultivated as previously described. Leaf tissues were harvested and immediately frozen in liquid nitrogen for total RNA extraction. For each sample, 1.5 μg of mRNA was used as input for library preparation. RNA sequencing libraries were constructed using the NEBNext Ultra RNA Library Prep Kit for Illumina (New England Biolabs), following the manufacturer’s instructions. RNA concentration was measured using a NanoDrop 2000C spectrophotometer (Thermo Scientific, Mississauga, Canada), and RNA integrity was assessed with an Agilent 2100 Bioanalyzer using an RNA Nano chip (Agilent Technologies, Santa Clara, CA, USA). RNA libraries were constructed and sequenced using the Illumina HiSeq 2500 platform at the Centre for Applied Genomics, SickKids Hospital (Toronto, Canada), under a fee-for-service agreement. Differential gene expression analysis between *MsDAD1*-OE lines and wild-type plants was performed using the DESeq2 R package (version 1.20.0). *P*-values were adjusted for multiple testing, and genes with an adjusted *p*-value< 0.001 were defined as differentially expressed genes (DEGs). Gene Ontology (GO) enrichment analysis was performed, and GO terms with a corrected *p*-value < 0.05 were considered significantly enriched. Functional annotation of DEGs was carried out using the NR, GO, and KEGG databases.

### 2.7 Statistical analysis

All experiments and gene expression analyses were conducted with at least three independent biological replicates. Results are presented as mean values ± standard error (SE). Statistical analyses were performed using one-way analysis of variance (ANOVA). Asterisks above columns indicate statistically significant differences compared to the control: *p* < 0.05 (*) and *p* < 0.01 (**). Different letters above histogram bars denote significant differences among treatments at *p* < 0.05, as determined by *post hoc* multiple comparison tests.

## 3 Results

### 3.1 The expression of *MsDAD1* was significantly induced under heat stress in alfalfa

**Figure 1:**
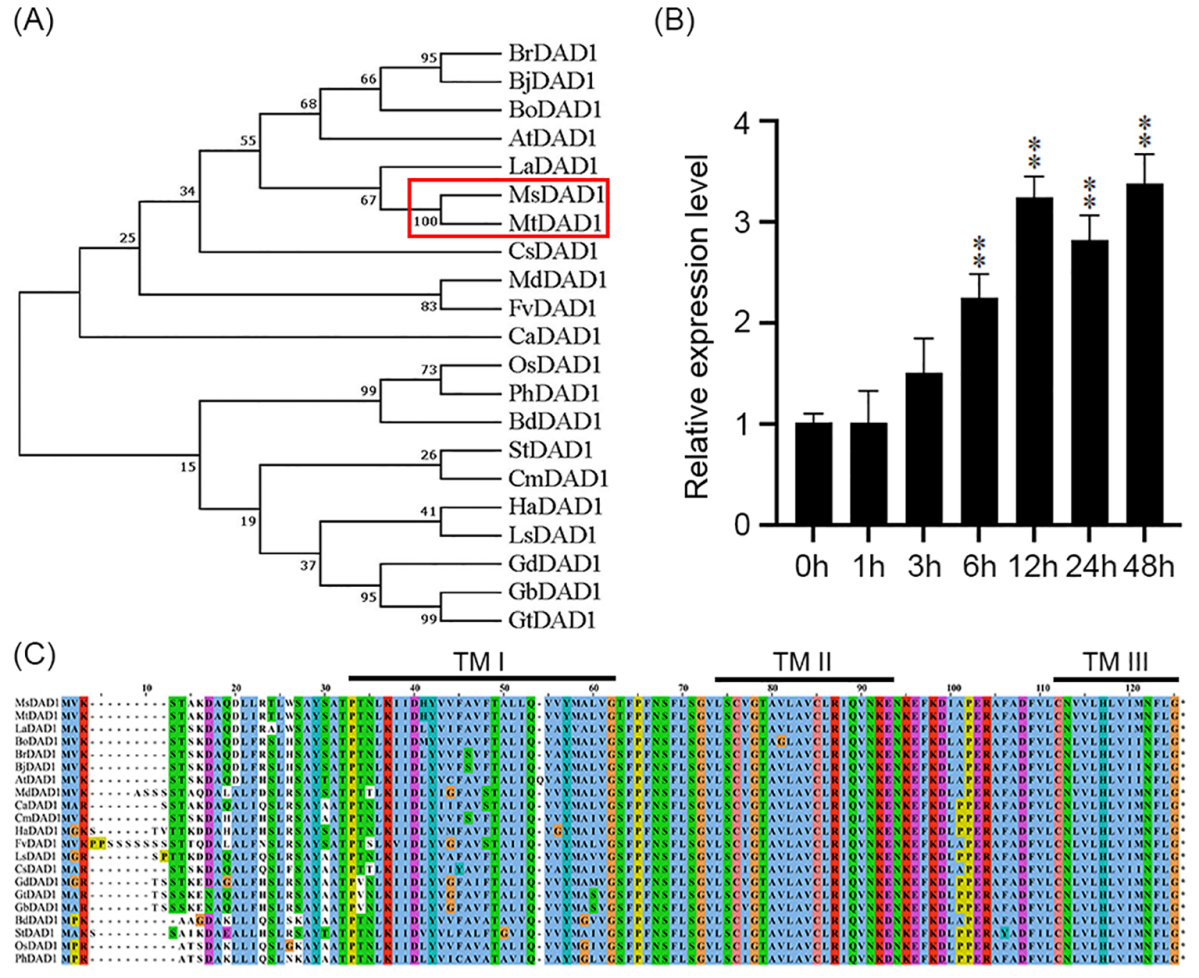
Sequence characteristics and heat stress response of *MsDAD1*. **(A)** Phylogenetic tree of *DAD1* homologs from various plant species constructed using the neighbor-joining (NJ) method based on amino acid sequences. Bootstrap values were calculated from 1000 replicates. **(B)** Expression profile of *MsDAD1* in alfalfa under heat stress (40°C) at different time points, as determined by qRT-PCR. Data represent mean ± SE of three biological replicates. **(C)** Conserved domain and structural features of *MsDAD1* and its homologs, highlighting predicted transmembrane (TM) domains and oligosaccharyltransferase (OST) subunit regions. **Asterisks indicate that the expression level of MsDAD1 in samples treated with high-temperature stress shows a significant difference (p < 0.01) compared with the 0 h control.

Based on transcriptomic analysis of alfalfa (*Medicago sativa* L.) from our previous study (Dong et al., 2018), a gene encoding *defender against apoptotic death 1* (*DAD1*), designated *MsDAD1* was identified. Phylogenetic analysis revealed that this gene shares the highest sequence homology with *MtDAD1* from *Medicago truncatula*, a model legume species, and was thus named *MsDAD1* (**Figure 1A**). Expression analysis showed that *MsDAD1* is significantly upregulated in response to heat stress (**Figure 1B**), suggesting its potential role in high-temperature adaptation. The coding sequence (CDS) of *MsDAD1* was subsequently cloned using primers listed in **Supplementary Table S1**. Consistent with other DAD1 orthologs, *MsDAD1* encodes a protein with three predicted transmembrane (TM) domains (TM I/II/III) and contains a conserved oligosaccharyltransferase (OST) subunit domain (**Figure 1C**). Phylogenetic analysis further demonstrated that *MsDAD1* shares 98%, 92%, and 87% sequence identity with DAD1 orthologs from *M. truncatula*, soybean (*Glycine max*), rice (*Oryza sativa*), and *Arabidopsis thaliana*, respectively, indicating that *DAD1* is highly conserved across plant species (**Figure 1C**; **Supplementary Table S1**).

### 3.2 Overexpression of *MsDAD1* enhanced heat stress tolerance in alfalfa

**Figure 2:**
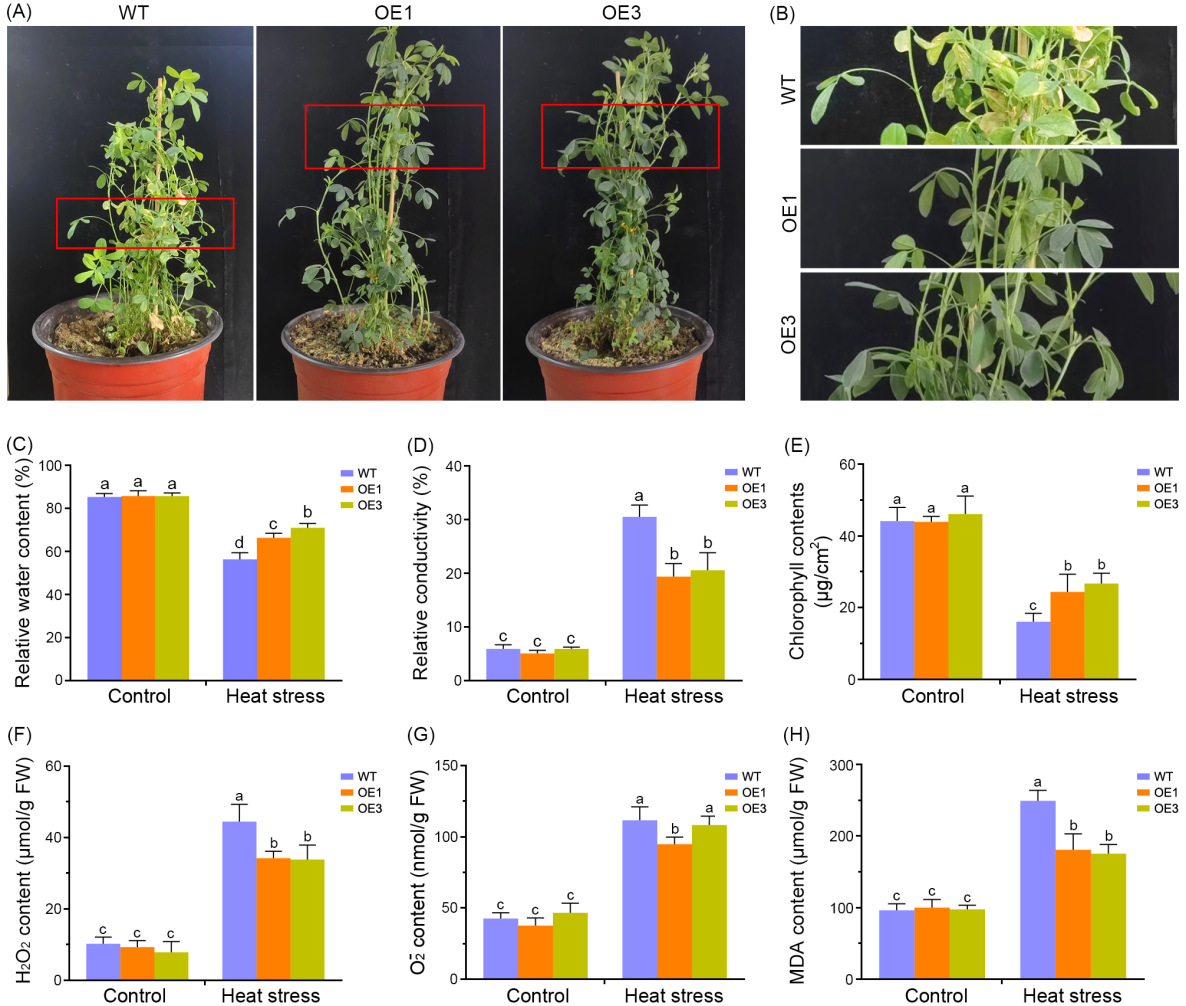
Overexpression of *MsDAD1* enhances heat tolerance in alfalfa. **(A)** Phenotypic comparison of wild-type (WT) and *MsDAD1*-overexpressing (OE) lines after exposure to high-temperature stress (35°C night/40°C day) for six days. **(B)** Enlarged view of the red-boxed region from panel **(A)**, highlighting leaf morphology differences. **(C-H)** Physiological responses of WT and *MsDAD1*-OE plants under control and heat stress conditions: **(C)** Relative water content (RWC), **(D)** Electrolyte leakage, **(E)** Chlorophyll content, **(F)** Hydrogen peroxide (H_2_O_2_) content, **(G)** Superoxide anion (O_2_^-^) content, **(H)** Malondialdehyde (MDA) content.

To investigate the role of *MsDAD1* in heat stress tolerance, transgenic alfalfa plants constitutively expressing *MsDAD1* were developed through stable genetic transformation. qRT-PCR analysis of six independent transgenic lines revealed that *MsDAD1*-OE1 and *MsDAD1*-OE3 exhibited the highest transcript levels and were selected for subsequent experiments (**Supplementary Figure S1**). Phenotypic evaluation under heat stress showed that *MsDAD1*-OE plants were more resilient than wild-type (WT) plants. While WT plants displayed pronounced leaf chlorosis and abscission, *MsDAD1*-OE lines maintained greener, healthier foliage with reduced visible damage (**Figures 2A, B**).

Physiological responses of WT and *MsDAD1*-OE lines were further evaluated under both normal and heat stress conditions. Under non-stress conditions, no significant differences in relative water content (RWC) were observed between the genotypes (**Figure 2C**). However, upon exposure to heat stress, RWC declined in all plants, with *MsDAD1*-OE lines maintaining significantly higher RWC compared to WT (**Figure 2C**). Electrolyte leakage, assessed via ion conductivity, was significantly elevated in both genotypes under heat stress, but the increase was more pronounced in WT plants, indicating greater membrane damage (**Figure 2D**). Correspondingly, *MsDAD1*-OE lines retained higher chlorophyll content than WT under heat stress, consistent with delayed senescence phenotypes (**Figure 2E**).

As heat stress disrupts reactive oxygen species (ROS) homeostasis, the accumulation of hydrogen peroxide (H_2_O_2_), superoxide anion (O_2_^-^), and malondialdehyde (MDA) were quantified. Under heat stress, *MsDAD1*-OE lines exhibited significantly lower levels of H_2_O_2_ and O_2_^-^ compared to WT (**Figures 2F, G**). MDA content, a marker of lipid peroxidation and oxidative damage, was also markedly reduced in *MsDAD1*-OE plants relative to WT (**Figure 2H**). These results suggest that *MsDAD1* overexpression mitigates heat-induced oxidative damage by enhancing ROS scavenging capacity.

Collectively, these findings demonstrate that *MsDAD1*-OE lines outperform WT under heat stress, exhibiting enhanced physiological stability, reduced oxidative damage, and improved stress tolerance.

### 3.3 MsDAD1 regulates the biosynthesis of jasmonic acid and abscisic acid in alfalfa

**Figure 3:**
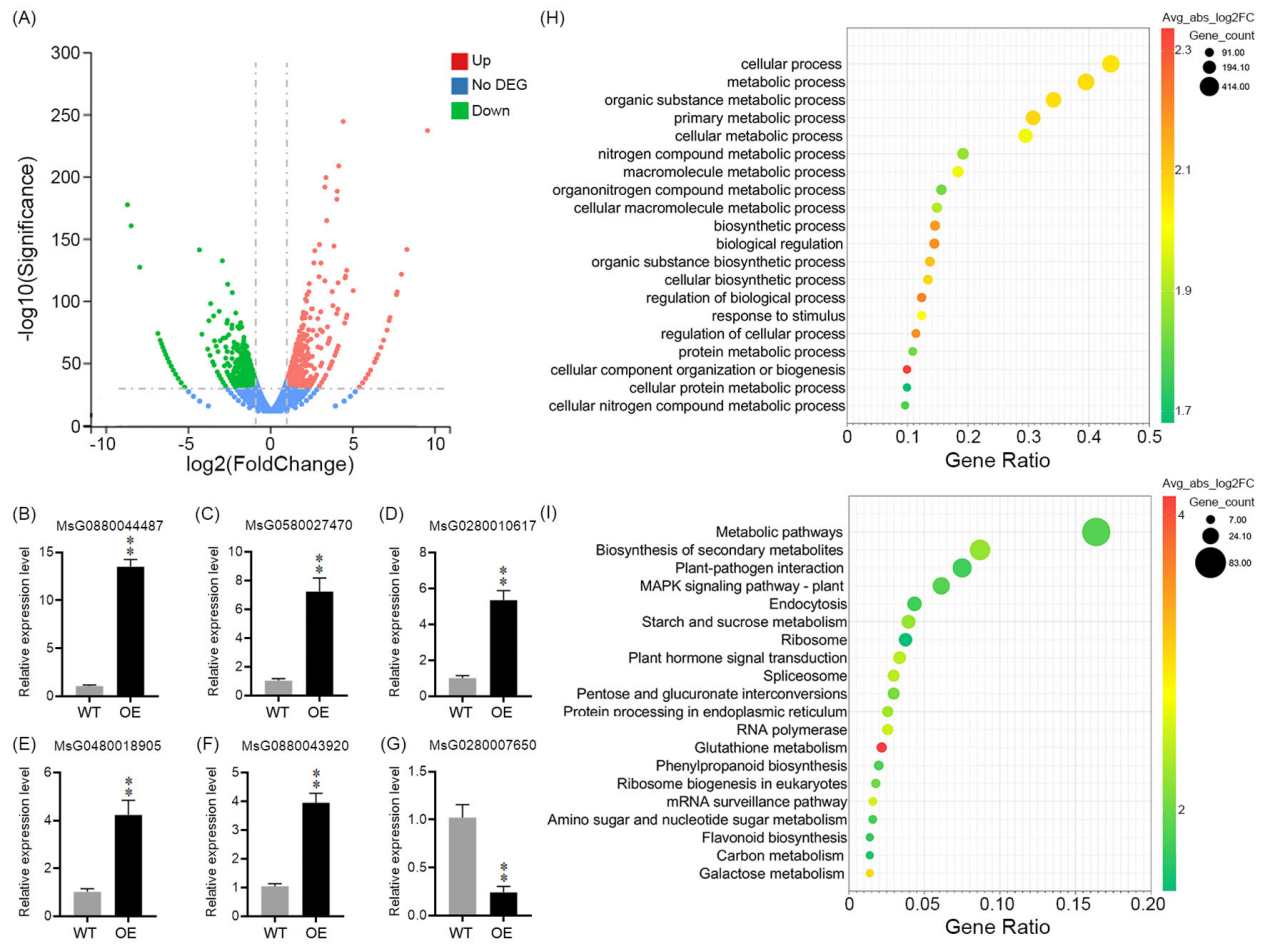
RNA-seq analysis of wild-type and *MsDAD1*-OE alfalfa seedlings. **(A)** Volcano plot showing differentially expressed genes (DEGs) between wild-type (WT) and *MsDAD1*-overexpressing (OE) seedlings. DEGs were defined by |Log_2_FoldChange| ≥ 1 and adjusted *p* < 0.05. **(B-G)** Validation of selected DEGs by qRT-PCR. Expression levels are shown relative to WT. Data represent mean ± SE from three biological replicates. **(H)** Gene Ontology (GO) enrichment analysis of DEGs. Significantly enriched GO terms are shown based on biological processes. **(I)** KEGG pathway enrichment analysis of DEGs. Dot size indicates the number of genes associated with each pathway, while color intensity reflects the adjusted *p*-value (*Padj*).

To elucidate the transcriptional changes regulated by MsDAD1, RNA-seq analysis on wild-type and *MsDAD1*-overexpressing (OE) alfalfa plants was performed. After quality control filtering, high-quality reads were retained for downstream analysis. A total of 1,088 differentially expressed genes (DEGs) were identified between WT and *MsDAD1*-OE lines, using thresholds of |Log_2_^FoldChange^| ≥ 1 and adjusted *p*-value < 0.05 (**Figure 3A**). Among these, 611 genes were upregulated and 477 genes were downregulated in *MsDAD1*-OE plants. qRT-PCR validation of six randomly selected genes confirmed the RNA-seq results, showing a high degree of consistency (**Figures 3B–G**).

Gene Ontology (GO) and Kyoto Encyclopedia of Genes and Genomes (KEGG) enrichment analyses revealed that DEGs were primarily associated with cellular and metabolic processes, biosynthesis of secondary metabolites, plant–pathogen interactions, MAPK signaling, plant hormone signal transduction, glutathione metabolism, and flavonoid and phenylpropanoid biosynthesis (**Figures 3H, I**; **Supplementary Tables S2**, **S3**).

**Figure 4:**
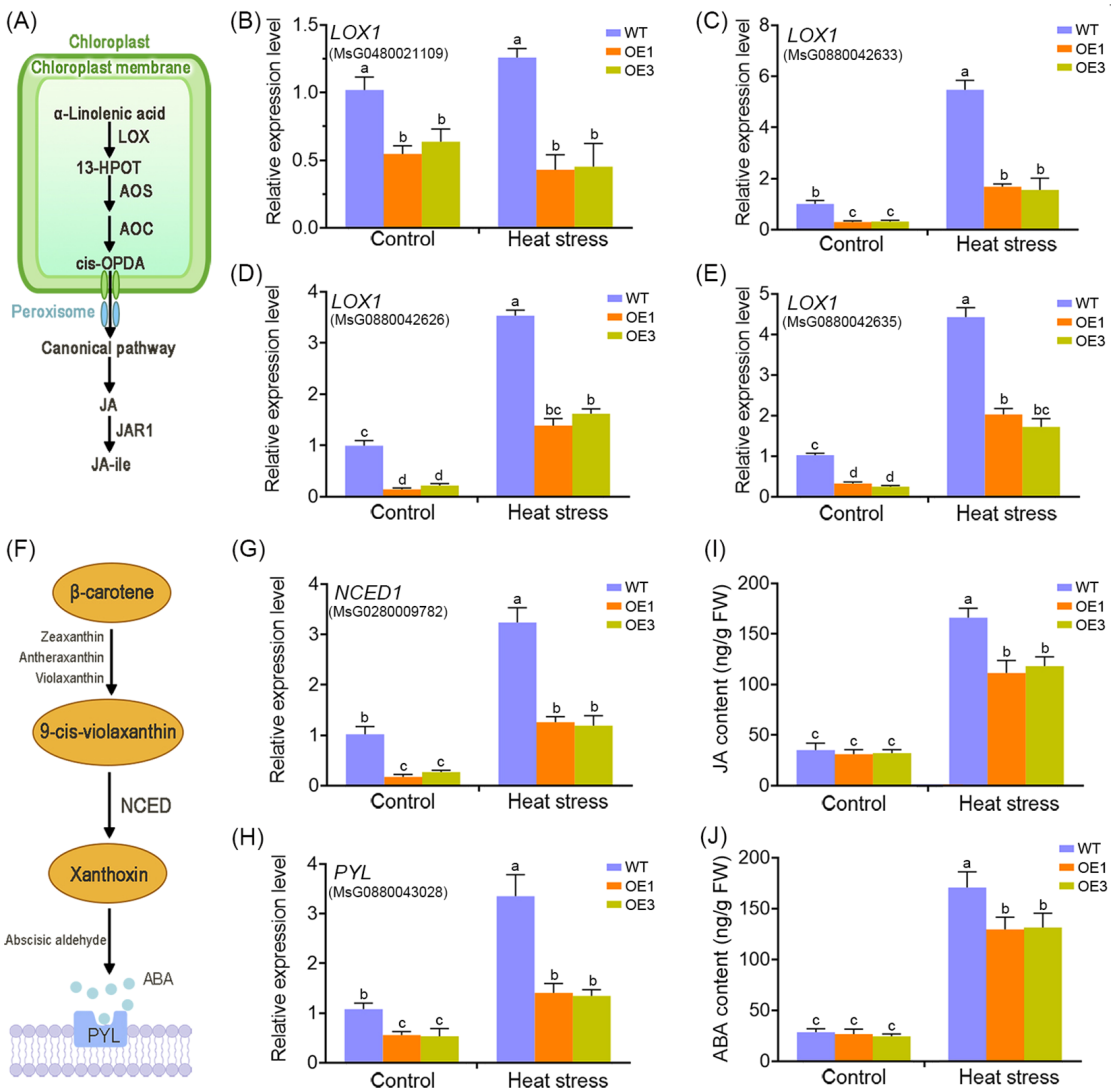
*MsDAD1* regulates the biosynthesis of jasmonic acid (JA) and abscisic acid (ABA) in alfalfa. **(A)** Simplified schematic representation of the JA biosynthetic pathway in plants. **(B-E)** Relative expression levels of *LOX1* in WT and *MsDAD1*-OE seedlings under normal and heat-stress conditions, as determined by qRT-PCR. **(F)** Simplified schematic representation of the ABA biosynthetic pathway. **(G, H)** Relative expression levels of *NCED1* (a key ABA biosynthesis gene) and *PYL* (an ABA receptor gene) in WT and *MsDAD1*-OE seedlings under normal and heat-stress conditions. **(I, J)** Endogenous levels of JA **(I)** and ABA **(J)** in WT and *MsDAD1*-OE seedlings under normal and heat-stress conditions.

Given the critical role of plant hormones in heat stress responses, we focused on hormone-related genes. Transcriptome data revealed that *MsDAD1*-OE plants exhibited reduced expression of key genes involved in jasmonic acid (JA) and abscisic acid (ABA) biosynthesis specifically, *LIPOXYGENASE1* (*LOX1*) and *9-cis-EPOXYCAROTENOID DIOXYGENASE1* (*NCED1*), respectively. This suggests that *MsDAD1* may modulate JA and ABA metabolism during heat stress. To validate this, the expression of *LOX1* and *NCED1* in WT and *MsDAD1*-OE plants were examined under control and heat stress conditions. Under normal conditions, both genes showed slightly lower expression in *MsDAD1*-OE plants compared to WT, but the differences were not statistically significant (*p* >0.05). Upon exposure to high temperatures, expression of both *LOX1* and *NCED1* was significantly upregulated in both genotypes; however, the induction was notably stronger in WT plants (**Figures 4B–E, G**). *LOX1* encodes a key enzyme in the JA biosynthetic pathway, catalyzing the conversion of α-linolenic acid to 13-hydroperoxyoctadecatrienoic acid (13-HPOT), a critical initial step in JA production (**Figure 4A**). *NCED1* is the rate-limiting enzyme in ABA biosynthesis, catalyzing the oxidative cleavage of carotenoids to produce xanthoxin, a precursor of ABA (**Figure 4F**). To determine whether altered gene expression translated into changes in hormone levels, endogenous JA and ABA concentrations under both control and heat stress conditions were quantified. Heat stress significantly increased the accumulation of both hormones in WT and *MsDAD1*-OE plants; however, the increase was significantly greater in WT plants (**Figures 4I, J**).

Together, these results indicate that *MsDAD1* negatively regulates the heat-induced accumulation of JA and ABA in alfalfa, likely through suppression of *LOX1* and *NCED1*, contributing to enhanced stress tolerance.

### 3.4 Ectopic expression of *MsDAD1* suppressed JA/ABA-induced leaf senescence

**Figure 5:**
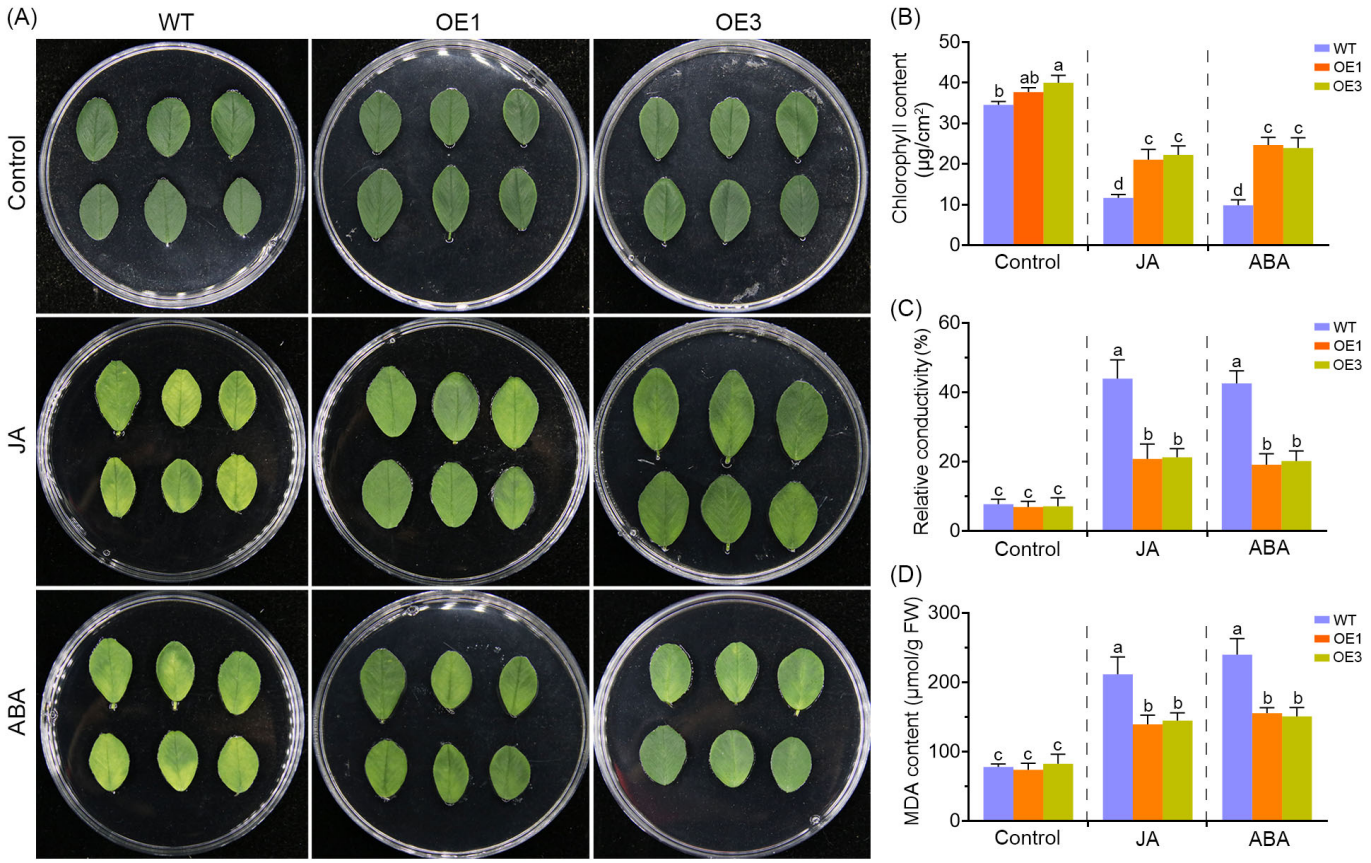
Overexpression of *MsDAD1* significantly suppresses JA- and ABA-induced leaf senescence. **(A)** Leaf senescence phenotypes of wild-type (WT) and *MsDAD1*-OE seedlings following treatment with control (mock), exogenous jasmonic acid (JA), or abscisic acid (ABA). **(B-D)** Quantification of chlorophyll content **(B)**, electrolyte leakage **(C)**, and malondialdehyde (MDA) content **(D)** in leaves corresponding to panel **(A)**.

To assess the involvement of JA and ABA in regulating leaf senescence in alfalfa, senescence phenotypes were evaluated following exogenous hormone treatments. Compared to the control, application of either JA or ABA significantly accelerated chlorophyll degradation, leaf yellowing, electrolyte leakage, and malondialdehyde (MDA) accumulation in both wild-type and *MsDAD1*-OE plants (**Figures 5A-D**). However, the severity of these senescence-associated responses was moderately attenuated in the *MsDAD1*-OE lines relative to wild-type plants. Under JA and ABA treatments, *MsDAD1*-OE plants retained higher chlorophyll content and exhibited lower electrolyte leakage and MDA levels compared to the wild type (**Figures 5B-D**). These findings suggest that *MsDAD1* delays the progression of leaf senescence by limiting excessive JA and ABA accumulation, particularly under heat stress conditions.

### 3.5 MsDAD1 is a key regulator involved in heat-mediated flowering in alfalfa

**Figure 6:**
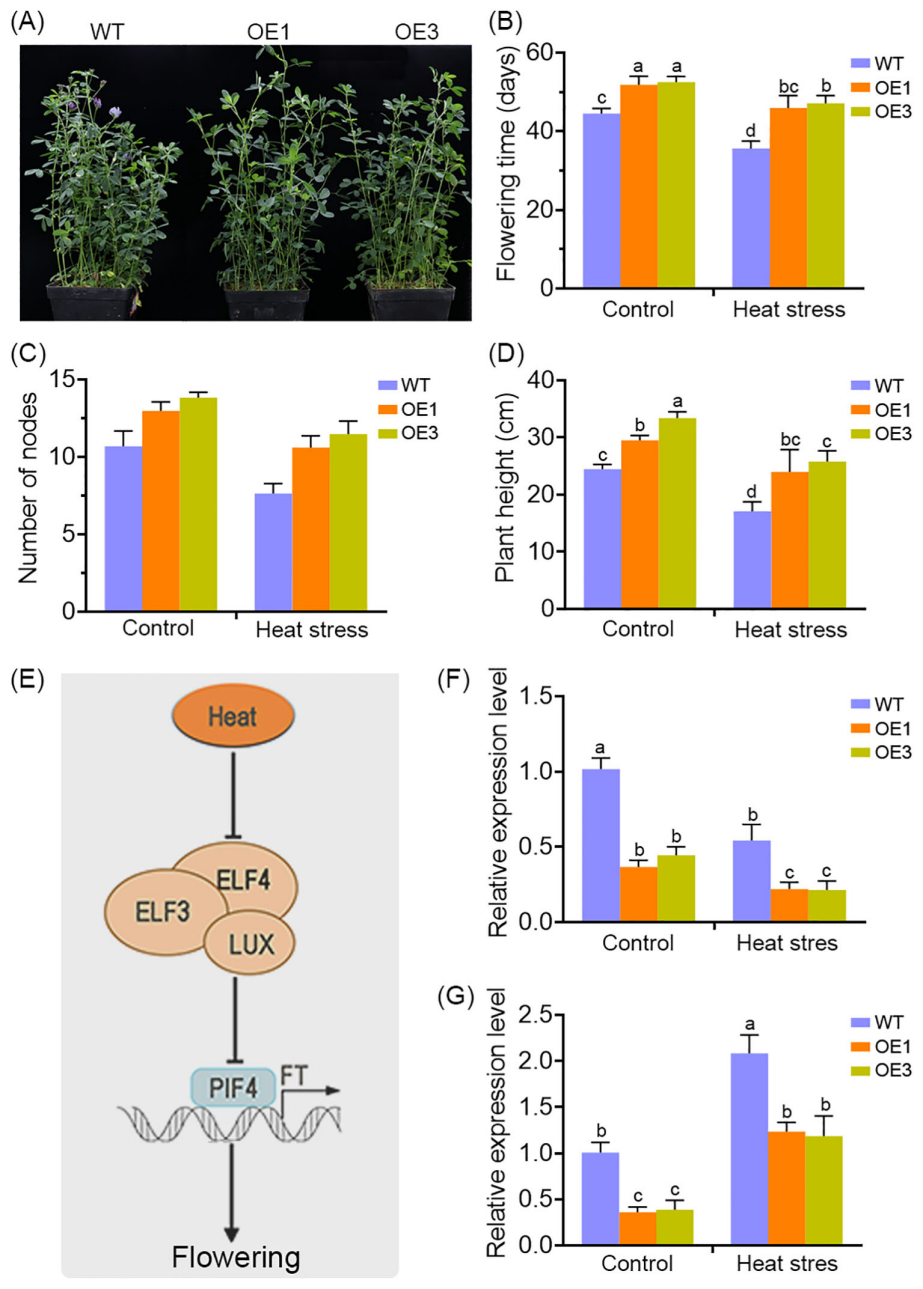
*MsDAD1* modulates heat stress-associated flowering in alfalfa. **(A)** Overexpression of *MsDAD1* delays flowering time in alfalfa under both normal and high-temperature conditions. **(B-D)** Quantification of flowering-related physiological traits in WT and *MsDAD1*-OE seedlings under control and heat stress conditions: **(B)** days to flowering, **(C)** node number at flowering, and **(D)** plant height at flowering. **(E)** Simplified schematic of the molecular module involved in heat stress-mediated flowering regulation, highlighting the roles of the evening complex (EC), PIF4, and *FT*. **(F, G)** Expression analysis of *ELF4* **(F)** and *FT* **(G)** in WT and *MsDAD1*-OE seedlings under control and heat stress conditions, as determined by qRT-PCR.

Extensive evidence indicates that heat stress can significantly disrupt the vegetative-to-reproductive transition in plants. In this study, longitudinal monitoring of developmental progression revealed a marked delay in flowering time in *MsDAD1*-OE lines compared to wild-type (WT) plants (**Figure 6A**). Under normal growth conditions, *MsDAD1*-OE plants exhibited delayed flowering, as reflected by a longer time to floral initiation, increased node number, and greater plant height at flowering onset (**Figures 6B–D**). Although high-temperature stress accelerated flowering in alfalfa, this effect was notably attenuated in *MsDAD1*-OE lines, suggesting a role for *MsDAD1* in modulating temperature-dependent flowering responses (**Figures 6B–D**).

Given the late-flowering phenotype of the *MsDAD1*-OE plants, the expression of flowering-related differentially expressed genes (DEGs) identified in our RNA-seq dataset were examined. Several key flowering regulators—such as *FLOWERING LOCUS T* (*FT*), *EARLY FLOWERING 4* (*ELF4*), and *FLOWERING TIME CONTROL GENE* (*FY*)—were downregulated in *MsDAD1*-OE plants (**Figure 6E**). These genes are known to play critical roles in regulating flowering under heat stress conditions.

To validate these observations, we analyzed the expression of *FT* and *ELF4* via qRT-PCR in both WT and *MsDAD1*-OE plants. Under normal conditions, expression levels of both genes were significantly lower in *MsDAD1*-OE lines compared to WT. Heat stress induced the expression of *FT* and *ELF4* in both genotypes, but the induction was significantly stronger in WT plants (**Figures 6F, G**). These results suggest that *MsDAD1* overexpression suppresses the heat-induced upregulation of flowering-promoting genes, thereby contributing to delayed flowering under elevated temperatures.

## 4 Discussion

Global warming has led to an increased frequency of extreme high-temperature events. Rising ambient temperatures driven by climate change are altering the geographical distribution of plant species and affecting a range of morphological and developmental traits, ultimately posing a serious threat to crop productivity (Matthews et al., 2019; Pausas, 2025). Alfalfa (*Medicago sativa* L.), though well-adapted to warm, semi-humid, and semi-arid environments, is particularly sensitive to high-temperature stress (Buzzanca et al., 2025). Exposure to heat combined with high humidity accelerates leaf senescence and triggers premature defoliation, significantly reducing both yield and forage quality (Arshad and Hannoufa, 2022).

In our previous transcriptomic analysis comparing salt-tolerant and salt-sensitive alfalfa genotypes, we identified *MsDAD1* and *MsDAD2* as salinity-induced genes (Dong et al., 2018). Functional studies revealed that overexpression of *MsDAD2* enhanced salt tolerance in transgenic alfalfa (Wang X  al., 2024). Despite sharing 95% sequence similarity, *MsDAD1* and *MsDAD2* display distinct stress-response profiles: *MsDAD1* is strongly induced by high-temperature stress, while *MsDAD2* is not. This divergence suggests that *MsDAD1* and *MsDAD2* may participate in separate abiotic stress signaling pathways.

The *DAD1* gene was originally identified in a temperature-sensitive mutant cell line (tsBN7) in animals, where it was shown to play a role in programmed cell death suppression (Zhou et al., 1997). *DAD1* encodes a subunit of the oligosaccharyltransferase (OST) complex, a central component of the endoplasmic reticulum (ER) machinery responsible for N-glycosylation of nascent proteins (Yan et al., 2005). This process involves the attachment of oligosaccharides to specific asparagine residues and is essential for correct protein folding and ER export (Kukuruzinska and Lennon, 1998). In plants, the functional roles of *DAD1* homologs under abiotic stress remain incompletely understood. For instance, *AtDAD1* has been shown to protect *Arabidopsis* protoplasts from UV-C-induced programmed cell death (PCD) (Danon et al., 2004), and in *Gladiolus* L., *DAD1* expression sharply declines during petal senescence (Yamada et al., 2004). However, the molecular mechanisms by which DAD1 proteins mediate stress responses and developmental processes remain largely uncharacterized.

*MsDAD1* suppresses the expression of key rate-limiting enzymes *LOX1* and *NCED1*, involved in jasmonic acid (JA) and abscisic acid (ABA) biosynthesis, respectively (**Figure 4**). This suppression was particularly pronounced under high-temperature stress conditions. JA and ABA are widely recognized as stress hormones involved in plant responses to both biotic and abiotic stressors (Wang et al., 2025). In addition to their roles in stress signaling, these hormones regulate several physiological processes, including root elongation, reproductive organ development, and senescence (Wan et al., 2025; Varshney and Majee, 2021; Kim et al., 2018). Previous studies have shown that heat shock activates the JA signaling pathway and promotes JA accumulation, as observed in agarwood and *Arabidopsis* through increased expression of biosynthetic genes such as *OPR3* (Wang et al., 2023; Xu et al., 2016; Tian et al., 2020). However, contrasting findings have been reported, suggesting a more nuanced role of JA in heat responses. For instance, Du et al. (2013) reported that genes involved in JA biosynthesis were downregulated under heat stress but upregulated during drought and cold stress. Similarly, Zhu et al. (2021) showed that elevated temperatures in *Arabidopsis* lead to reduced JA levels due to the upregulation of *JOXs* and *ST2A*, which degrade active JA. In cotton, high temperatures suppressed the expression of *GhAOC2* in anthers, leading to reduced JA biosynthesis (Khan et al., 2023). These seemingly contradictory findings may stem from differences in plant species, experimental designs, stress intensity, or exposure duration. Thus, JA levels are not static during heat stress but are influenced by multiple factors. Short-term or moderate heat stress may elevate JA levels to promote stress tolerance, whereas prolonged or extreme heat stress can lead to excessive JA and ABA accumulation, which may trigger premature senescence and cell death. In *Arabidopsis*, *OXI1* and *DAD1* were shown to antagonistically regulate light-induced cell death through modulation of JA and salicylic acid (SA) levels (Beaugelin et al., 2019). Furthermore, many studies have demonstrated that ABA and JA can act synergistically under environmental stress conditions (Wang et al., 2025). In *Arabidopsis* and tobacco, ABA receptor proteins such as PYRABACTIN RESISTANCE1-Like (PYLs) regulate metabolic reprogramming via the JA signaling pathway (Aleman et al., 2016). These findings point to a complex JA–ABA crosstalk network that fine-tunes plant metabolism and growth.

Whether *MsDAD1* participates directly in metabolic homeostasis or signaling crosstalk between JA and ABA remains to be determined. Future research is needed to elucidate how *MsDAD1* specifically responds to high-temperature stress and modulates JA and ABA biosynthesis or signaling. In addition to its role in hormone regulation, *MsDAD1* also appears to influence flowering time in alfalfa. Plants overexpressing *MsDAD1* exhibited delayed flowering under both normal and heat stress conditions (**Figures 6A-D**). Transcriptome profiling revealed significant downregulation of the flowering-time regulators *FLOWERING LOCUS T* (*FT*) and *EARLY FLOWERING 4* (*ELF4*), with a more pronounced effect under heat stress (**Figures 6E-G**). The role of *FT* in regulating flowering time is well established in various plant species, including alfalfa (Kang et al., 2019). In *Arabidopsis thaliana*, *ELF3* functions as a central component in temperature sensing and thermomorphogenesis by participating in the evening complex (EC), together with *ELF4* and *LUX ARRYTHMO* (*LUX*) (Zhu et al., 2023; Liu et al., 2024). Recent studies suggest that warm temperatures inhibit the EC complex’s DNA-binding activity by reducing the subnuclear localization of *ELF3*, thereby permitting *PIF4* to interact with *FT* and promote flowering (Preston and Fjellheim, 2022). This *EC–PIF4–FT* module represents a critical mechanism in temperature-regulated flowering. Based on our current findings, we propose that *MsDAD1* may modulate flowering time in alfalfa through regulation of the *EC–PIF4–FT* signaling axis. However, the precise molecular mechanism by which *MsDAD1* interfaces with this pathway remains largely unexplored and warrants further investigation.

## Data Availability

The original contributions presented in the study are included in the article/**Supplementary Material**. Further inquiries can be directed to the corresponding author.

## Supplementary material

Supplementary Figure 1 qRT-PCR analysis of *MsDAD1* in wild type and transgenic seedlings.

Supplementary Table 1 List of primers used in this study.

Supplementary Table 2 GO analysis of the DEGs between the wild-type and *MsDAD1*-OE line.

Supplementary Table 3 KEGG analysis of DEGs between the wild-type and *MsDAD1*-OE line.
