# Supplementary figures and images for: MsDAD1 acts as a heat-induced “senescence brake” in alfalfa
Source: Front Plant Sci. 2025 Sep 5;16:1664465. doi: 10.3389/fpls.2025.1664465 (PMC12447589; doi:10.3389/fpls.2025.1664465)

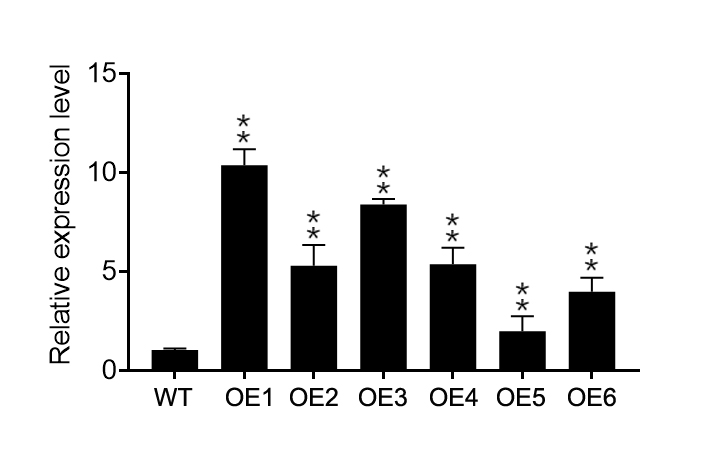

Supplement: Supplementary Figure 1 — qRT-PCR analysis of MsDAD1 in wild type and transgenic seedlings. [file Image1.jpeg]
