# Supplementary material for: MsDAD1 acts as a heat-induced “senescence brake” in alfalfa
Source: Front Plant Sci. 2025 Sep 5;16:1664465. doi: 10.3389/fpls.2025.1664465 (PMC12447589; doi:10.3389/fpls.2025.1664465)
Supplement: Supplementary Table 2 — GO analysis of the DEGs between the wild-type and MsDAD1-OE line. [file Table2.docx]

>MsDAD1,*Medicago sativa*

MVKSTAKDAQDLLRTLWSAYSATPTNLKIIDHYVVFAVFTALLQVVYMALVGTFPFNSFLSGVLSCVGTAVLAVCLRIQVNKENKEFKDIAPERAFADFVLCNVVLHLVIMNFLG*

>MtDAD1, *Medicago truncatula*

MVKSTAKDAQDLIRTLWSAYSATPTNLKIIDHYVVFAVFTALIQVVYMALVGTFPFNSFLSGVLSCVGTAVLAVCLRIQVNKENKEFKDIAPERAFADFVLCNVVLHLVIMNFLG*

>BdDAD1, *Brachypodium distachyon*

MPKAAGDAKLLIQSLSKAYAATPTNLKIIDLYVVFAVATAVVQVVYMGVVGSFPFNSFLSGVLSCIGTAVLAVCLRIQVNKDNKEFKDLAPERAFADFVLCNLVLHLVIMNFLG*

>OsDAD1, *Oryza sativa*

MPRATSDAKLLIQSLGKAYAATPTNLKIIDLYVVFAVATALIQVVYMGIVGSFPFNSFLSGVLSCIGTAVLAVCLRIQVNKDNKEFKDLPPERAFADFVLCNLVLHLVIMNFLG*

>PhDAD1, *Petunia hybrida*

MPRATSDAKLLIQSLNKAYAATPTNLKIIDLYVICAVATAVIQVAYMGLVGSFPFNSFLSGVLSCIGTAVLAVCLRIQVNKDNKEFKDLPPERAFADFVLCNLVLHLVIMNFLG*

>LaDAD1, *Lupinus albus*

MAKSTSKDAQDLFRALWSAYSATPTNLKIIDLYVIFAVFTALIQVAYVALVGSFPFNSFLSGVLSCVGTAVLAVCLRIQVNKENKEFKDLAPERAFADFVLCNVVLHLVIMNFLG*

>GbDAD1, *Gossypium barbadense*

MARTSSSKENAQALFHSLRSAYAATPVNLKIIDLYVGFAVFTALIQVVYMASVGSFPFNSFLSGVLSCVGTAVLAVCLRIQVNKENKEFKDLPPERAFADFVLCNLVLHLVIMNFLG*

>GdDAD1, *Gossypium darwinii*

MGRTSSTKEDAGALFHSLRSAYAATPVNLKIIDLYVGFAIFTALIQVVYMAMVGSFPFNSFLSGVLSCVGTAVLAVCLRIQVNKENKEFKDLPPERAFADFVLCNLVLHLVIMNFLG*

>GtDAD1, *Gossypium tomentosum*

MARTSSSKENAQALFHSLRSAYAATPVNLKIIDLYVGFAVFTALIQVVYMASVGSFPFNSFLSGVLSCVGTAVLAVCLRIQVNKENKEFKDLPPERAFADFVLCNLVLHLVIMNFLG*

>StDAD1, *Solanum tuberosum*

MAKSSAIKDAEALLHSLRSAYTATPTNLKIIDVYVLFALFTGVIQVVYMAIVGSFPFNSFLSGVLSCIGTAVLAVCLRIQVNKENKEFKDLPPERAYADFILCNLVLHLVIMNFLG*

>AtDAD1, *Arabidopsis thaliana*

MVKSTSKDAQDLFHSLHSAYTATPTNLKIIDLYVCFAVFTALIQQVAYMALVGSFPFNSFLSGVLSCIGTAVLAVCLRIQVNKENKEFKDLAPERAFADFVLCNLVLHLVIINFLG*

>BoDAD1, *Brassica oleracea*

MVKSTSKDAQDLFRSLHSAYSATPTNLKIIDMYVVFAVFTALIQVAYMALVGSFPFNSFLSGVLSCVGTAGLAVCLRIQVNKENKEFKDLAPERAFADFVLCNLVLHLVIINFLG*

>BrDAD1, *Brassica rapa*

MVKSTSKDAQDLFRSLHSAYSATPTNLKIIDLYVVFSVFTALIQVAYMALVGSFPFNSFLSGVLSCIGTAVLAVCLRIQVNKENKEFKDLAPERAFADFVLCNLVLHLVIINFLG*

>BjDAD1, *Brassica juncea*

MVKSTSKDAQDLFRSLHSAYSATPTNLKIIDLYVVFSVFTALIQVAYMALVGSFPFNSFLSGVLSCIGTAVLAVCLRIQVNKENKEFKDLAPERAFADFVLCNLVLHLVIINFLG*

>MdDAD1, *Malus domestica*

MVKASSSSTAQDALALFDSLRSAYSATPTTLKIIDLYIGFAVSTALIQVVYMALVGSFPFNSFLSGVLSCVGTAVLAVCLRIQVNKENKEFKDLAPERAFADFVLCNVVLHLVIMNFLG*

>FvDAD1, *Fragaria vesca*

MVKPPSSSSSSSSTTQDALALFNSLRSAYSATPTSLKVIDLYVGFAVSTAIIQVVYMALVGSFPFNSFLSGVLSCIGTAVLAVCLRIQVNKENKEFKDLAPERAFADFVLCNLVLHLVIMNFLG*

>CsDAD1, *Cucumis sativus*

MARSTSKDAQALFQSLFSAYAATPTTLKIIDLYVIYAVFTALIQVAYMAIVGSFPFNSFLSGVLSCIGTAVLAVCLRIQVNKENKEFKDLAPERAFADFVLCNLVLHLVIINFLG*

>HaDAD1, *Helianthus annuus*

MGKSTVTTKDDAHALFHSLRSAYSATPTNLKIIDLYVMFAVFTALIQVGYMAIVGSFPFNSFLSGVLSCVGTAVLAVCLRIQVNKENKEFKDLPPERAFADFVLCNLVLHLVIMNFLG*

>LsDAD1, *Lactuca sativa*

MGRSPTTKDDAQALFQSLRSAYAATPTNLKIIDLYVAFAVFTAVIQVVYMAVVGSFPFNSFLSGVLSCVGTAVLAVCLRIQVNKENKEFKDLPPERAFADFVLCNLVLHLVIMNFLG*

>CmDAD1, *Cucumis melo*

MVKSTSKDAHALFHSLRSAYAATPTNLKIIDLYVVFSVFTALIQVVYMAIVGSFPFNSFLSGVLSCIGTAVLAVCLRIQLNKENKEFKDLPPERAFADFVLCNLVLHLVIMNFLG*

>CaDAD1, *Capsicum annuum*

MARSSTAKDAQALIQSLRSAYAATPTNLKIIDLYIIFAVSTALIQVVYMALVGSFPFNSFLSGVLSCVGTAVLAVCLRIQVNKENKEFKDLPPERAFADFVLCNLVLHLVIMNFLG*
